# Mowat–Wilson Syndrome: Case Report and Review of *ZEB2* Gene Variant Types, Protein Defects and Molecular Interactions

**DOI:** 10.3390/ijms25052838

**Published:** 2024-02-29

**Authors:** Caroline St. Peter, Waheeda A. Hossain, Scott Lovell, Syed K. Rafi, Merlin G. Butler

**Affiliations:** 1 Departments of Psychiatry & Behavioral Sciences and Pediatrics, University of Kansas Medical Center, 3901 Rainbow Blvd. MS 4015, Kansas City, KS 66160, USA; c527s208@kumc.edu (C.S.P.); whossain@kumc.edu (W.A.H.); rafigene@yahoo.com (S.K.R.); 2 Protein Structure Laboratory, University of Kansas, Lawrence, KS 66047, USA; swlovell@ku.edu

**Keywords:** Mowat–Wilson syndrome (MWS), case report, review, *ZEB2* gene variants, ZEB2 protein domains and defects, ZEB2 functional molecular interactions

## Abstract

Mowat–Wilson syndrome (MWS) is a rare genetic neurodevelopmental congenital disorder associated with various defects of the zinc finger E-box binding homeobox 2 (*ZEB2*) gene. The *ZEB2* gene is autosomal dominant and encodes six protein domains including the SMAD-binding protein, which functions as a transcriptional corepressor involved in the conversion of neuroepithelial cells in early brain development and as a mediator of trophoblast differentiation. This review summarizes reported *ZEB2* gene variants, their types, and frequencies among the 10 exons of *ZEB2*. Additionally, we summarized their corresponding encoded protein defects including the most common variant, c.2083 C>T in exon 8, which directly impacts the homeodomain (HD) protein domain. This single defect was found in 11% of the 298 reported patients with MWS. This review demonstrates that exon 8 encodes at least three of the six protein domains and accounts for 66% (198/298) of the variants identified. More than 90% of the defects were due to nonsense or frameshift changes. We show examples of protein modeling changes that occurred as a result of *ZEB2* gene defects. We also report a novel pathogenic variant in exon 8 in a 5-year-old female proband with MWS. This review further explores other genes predicted to be interacting with the *ZEB2* gene and their predicted gene–gene molecular interactions with protein binding effects on embryonic multi-system development such as craniofacial, spine, brain, kidney, cardiovascular, and hematopoiesis.

## 1. Introduction

Rare genetic disorders have been estimated to affect up to 10% of the population [1]. Advances in genomic technologies such as exome sequencing are becoming more widely used to gain insight into the cause and diagnosis of these rare disorders by identifying specific molecular defects and outcomes in patients with Mendelian or non-Mendelian disorders. Exome sequencing studies have shown underlying causative genes in approximately 25% of cases [2]. A growing number of gene variants and types involved in protein production have not been well characterized in rare disorders, including Mowat–Wilson syndrome.

Mowat–Wilson syndrome (MWS) is an example of a rare genetic disorder with intellectual disabilities with multiple congenital anomalies and multi-system involvement. This disorder was first reported in 1998 [3] and now about 300 patients have been found in the medical literature or databases. Chromosome 2q22-2q23 deletions were reported in the first patients with this disorder and the zinc finger E-box binding homeobox 2 (*ZEB2*; NM_014795.4) gene was found in this region. Other patients with MWS with overlapping features having *ZEB2* gene deletions or duplications of different sizes and intragenic variants have been identified [4,5,6].

Mowat–Wilson syndrome shows clinical variability and is recognized as a multiple congenital anomaly disorder involving several organ systems. Clinical facial features include a square-shaped face with a prominent and triangular chin, high forehead, large eyebrows with medial flaring, hypertelorism, deep-set and large eyes, broad and depressed nasal bridge, rounded nasal tip, prominent columella, open mouth, and M-shaped upper lip. The ears are posteriorly rotated with large, uplifted earlobes with a central depression reminiscent of a red blood corpuscle. The face lengthens with age and the chin becomes more prominent with the appearance of broad-appearing eyebrows. Individuals with this disorder often have severe intellectual disability with a mean age of walking at four years and a wide-based gait with a tendency for flexed arms in resting position [7,8,9,10]. Most individuals with MWS have seizures (84%) and an abnormal EEG. Short stature and microcephaly are often present with cerebral anomalies including corpus callosum and hippocampal defects, enlargement of cerebral ventricles, white matter abnormalities, large basal ganglia, and cortical and cerebellar malformations. Gastrointestinal problems such as chronic constipation are present and are most often related to lack of innervation causing Hirschsprung disease of either the short or long segment variety and are documented in about 50% of patients. Congenital heart disease is reported in 58% of patients including patent ductus arteriosus, atrial septal or ventricular septal defects, pulmonary stenosis, aortic coarctation, Tetralogy of Fallot, aortic valve abnormalities, and a pulmonary artery sling with or without tracheal stenosis/hypoplasia. Genitourinary and kidney anomalies are common including hypospadias, bifid scrotum, cryptorchidism, pelvic or duplex kidneys, and hydronephrosis [7,8]. The wide range of clinical findings may relate to different *ZEB2* gene variants and therefore additional research is needed to identify the type and frequency of gene variants and their impact on protein structure and function.

The *ZEB2* gene is located on chromosome 2q22.3 and expressed in the human nervous system throughout development, exemplifying its importance in gliogenic and neurogenic processes. ZEB2 has been documented to play roles in the induction of the neuroectoderm and neural crest. It acts to direct neural crest cells and regulates the development of cerebral regions, along with development of the spinal cord, cardiac, and enteric systems [8,9]. Hence, individuals with MWS can present with a combination of multi-system deficits with variable penetrance [10,11,12].

In searching the medical literature and unreported databases, we found a total of 298 patients with MWS and *ZEB2* variants. We tabulated these variants in the *ZEB2* gene and protein and summarized the frequency of gene variants within each of the 10 exons and their relationship to protein structure and function. We also obtained the predicted *ZEB2* gene interactions with other genes implicated and molecular pathways pertaining to neurodevelopmental multi-system involvement. Additionally, we described a new patient with MWS having a novel pathogenic variant due to a heterozygous c.2471_2475del5 in exon 8 of the *ZEB2* gene.

## 2. Detailed Case Description

### 2.1. Clinical Case Report

Our proband was prenatally diagnosed with a complex cardiovascular single ventricular disorder and subsequent amniocentesis genetic testing was normal. She was delivered at 37 weeks’ gestation with a double outlet right ventricle, subaortic and anterior muscular ventricular septal defects, and hypoplastic mitral and tricuspid valves with a hypoplastic left heart. The family initially took her home for palliative care, but upon further evaluation, underwent multiple cardiac and surgical procedures, including Fontan and Glenn procedures. These surgical interventions, which took place between one and six months of age, led to a partial recovery of cardiac function.

Our proband has two healthy siblings without cardiac or other disorders. The family history was also unremarkable for birth defects and no consanguinity was noted. The patient required G-tube feedings and close health monitoring with multiple evaluations and hospitalizations throughout infancy. She had a normal karyotype and chromosomal microarray studies during infancy.

Around one year of age, a comprehensive connective tissue genetic test including the autosomal dominant *ZEB2* gene (NM_014795) was ordered via a commercially approved genetic testing laboratory (Connective Tissue Gene Tests (CTGT)). The DNA sequencing revealed a heterozygous c.2471_2475del5 in exon 8 of the *ZEB2* gene. These five base pair deletions resulted in a frameshift, causing aberrant mRNA transcription and the defective protein causing MWS. All coding exons and exon boundaries of the gene were amplified by PCR and ABI 3730 sequencers, as standard genetic testing at the time. Additionally, coding exons and exon boundaries were analyzed for copy number variation using a high-density targeted array. The genetic defect was considered de novo in view of the negative family history, including two unaffected siblings for birth defects or features of MWS. *ZEB2* is an autosomal dominant gene and parental DNA testing was not undertaken.

**Figure 1:**
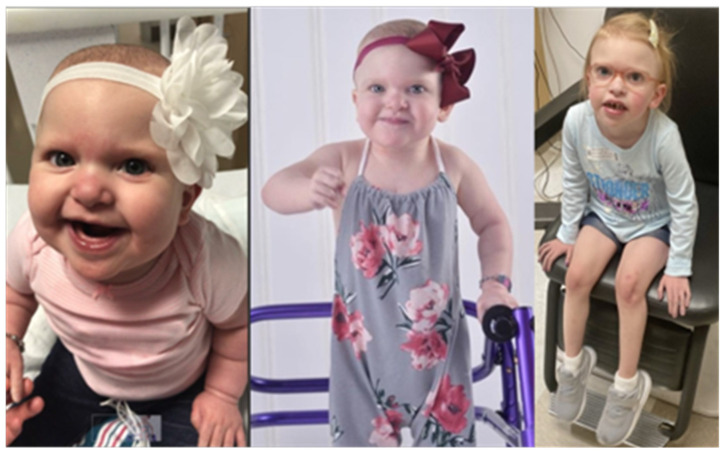
Our proband with typical craniofacial features and phenotype of MWS. She had a pathogenic heterozygous c.2471_2475del5 in exon 8 of the *ZEB2* gene. Photos were obtained with consent during infancy, early childhood, and before her death at about five years of age.

At about three years of age her height was 87.3 cm (6%ile), weight was 11.7 kg (7%ile), and body mass index was 15.35 kg/m^2^ (36%ile). At that age, her heart rate was 120, respiration was 20, oxygen saturation was 80%, blood pressure (right arm) was 85/33, and blood pressure (right leg) was 107/54. At five years and four months of age, her height was 102.1 cm (3%ile), weight was 14.3 kg (3%ile), and body mass index was 13.72 kg/m^2^ (15%ile). She was able to write, recognize a few written words, and perform several preschool-appropriate skills. She spoke at the level of a two-and-a-half-year-old and communicated her wants and needs, both verbally and with sign language. She had severe cardiac defects, including, but not limited to hypoplastic left heart syndrome (HLHS), ventricular septal defects (VSDs), left pulmonary artery (LPA) sling causing tracheomalacia, dysplastic tricuspid valve causing severe tricuspid regurgitation (TR), and partial anomalous pulmonary venous connection (PAPVR). She presented with typical MWS facial features, a duplex kidney and VUR, visual deficits, tooth abnormalities, hypotonia, global delays, secondary kidney and liver issues, high intracranial pressure (pseudotumor cerebri), and GI issues. She was not able to eat food, as she would develop severe abdominal pain, intractable vomiting, GI bleeding, and colitis, often requiring hospitalization for several weeks requiring IV fluids and/or TPN after ingestion of any amount of food. She was treated with diuretics. She was G-tube dependent and fed neonate infant formula (an elemental formula), with some GI bleeding, vomiting, and discomfort noted at baseline. Although Hirschsprung disease was ruled out based on rectal biopsy studies, she had gastroparesis and chronic constipation. Her EEG studies on more than one occasion were normal. She had no documented seizures/epilepsy (see Figure 1). Her overall health status, function, and quality of life continued to decline in spite of continuous care and monitoring. Her parents made the ultimate decision to enroll her in hospice care where she passed away at about 5 years of age.

### 2.2. Genetic and Protein Domain Data Collection of Patients with Mowat-Wilson Syndrome

Computer literature and unreported databases were searched for keywords such as Mowat–Wilson, *ZEB2* gene and protein defects or variants, or clinical features using PUBMED (www.pubmed.com; accessed on 1 October 2022) and performed from 2001 to the present (2023). About 180 published reports were found with the most useful data obtained from approximately 20 articles, as summarized in Table 1. From this study, we analyzed 266 cases of Mowat–Wilson syndrome and an additional 32 deidentified unpublished patients accessed from the Mowat–Wilson Syndrome Foundation for a total of 298 patients. These sources were used to collect data regarding *ZEB2* gene variants, types, frequencies, and protein defects along with domain locations and functions. We found that exon 8 encodes at least three of the six protein domains of the *ZEB2* gene and accounts for 66% (198/298) of the variants identified.

### 2.3. ZEB2 Gene Variant Types and Frequencies

**Figure 2:**
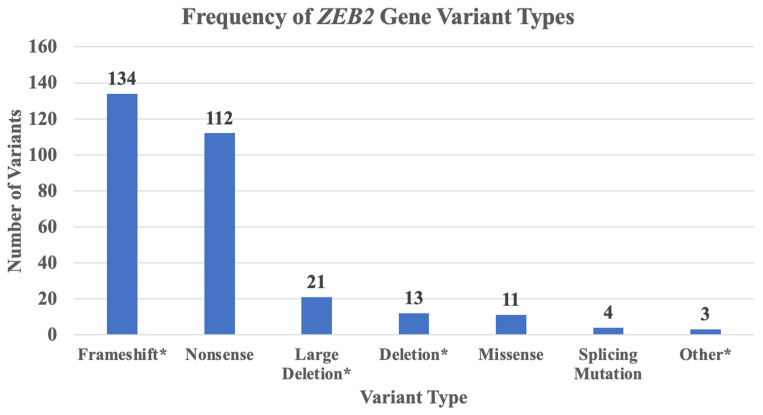
Frequency of *ZEB2* gene variants identified by variant type in 298 reported patients with MWS. The frequencies of *ZEB2* gene variants were identified and grouped. Frameshift* represents a combination of frameshift alone (N = 88), and frameshift plus variants such as frameshift with small deletion (N = 29), frameshift with small insertion (N = 13), or frameshift with small indel (N = 4). Large deletion* represents a combination of large deletions (N = 15) and chromosome deletions (N = 6). Deletion* represents a combination of deletions involving DNA (N = 9) or whole exome based (N = 4). Other* represents a combination of in-frame defects with intragenic deletion (N = 1), partial duplication (N = 1) or insertion deletion (N = 1).

*ZEB2* gene variants, types, and frequencies were analyzed from 298 studied individuals with MWS and summarized in Figure 2. The most prevalent variant type was frameshift (134 patients; 45%) followed by nonsense (112 patients; 38%).

**Figure 3:**
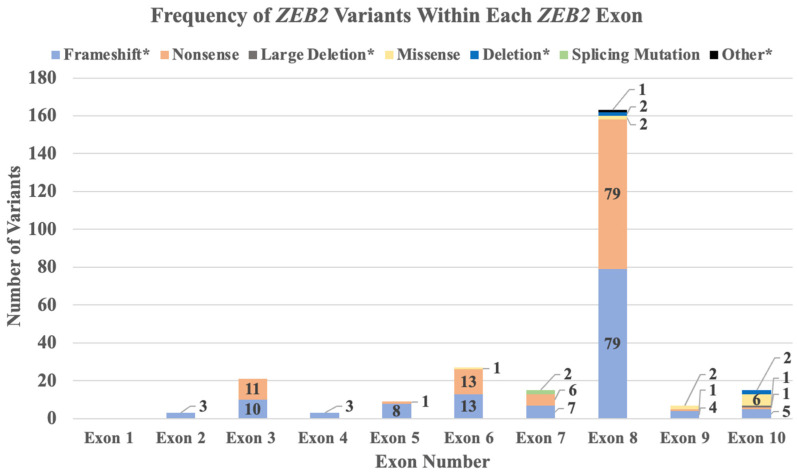
Number of *ZEB2* gene variants and types identified in each exon.

Of the 298 patients with MWS, 262 had sufficient information to determine the *ZEB2* exon variant type and to analyze the distribution and frequency of *ZEB2* variants within a specific exon. Figure 3 represents the exons within the gene, along with the frequency and type of variants within each exon. The most common variant site or location was c.2083C>T within exon 8 of the *ZEB2* gene. This variant was found in 11 percent of all patients reported with MWS.

### 2.4. ZEB2 Gene–Gene or Protein Interactions

**Figure 4:**
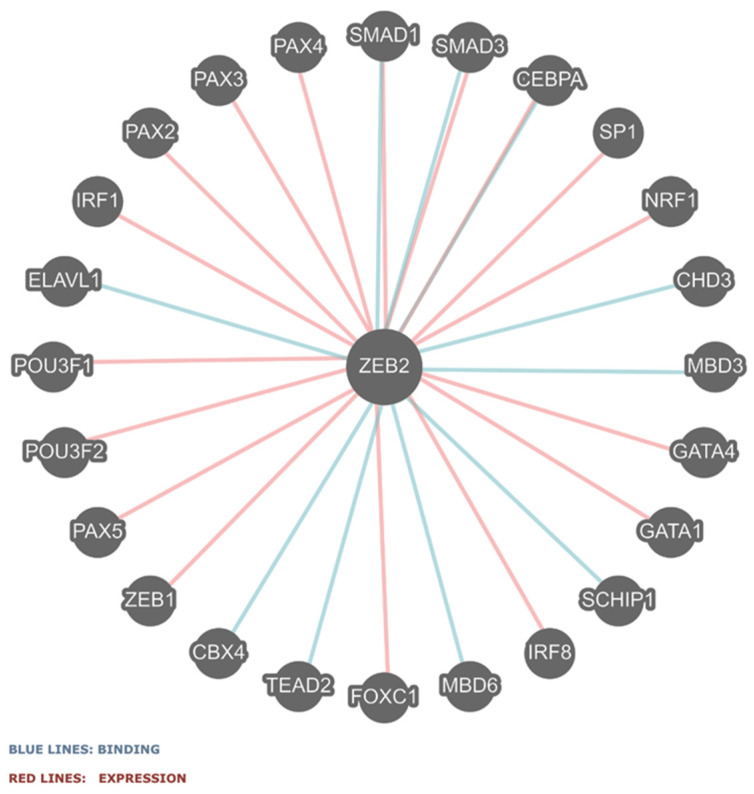
*ZEB2* gene–gene functional interactions are identified via binding (blue lines) and co-expression (red lines) [http://pathwaycommons.org/pc12/Pathway_751ce7ccec6e191682a33d7252aac8] (accessed on 1 November 2023). These gene interactions relate to TGF receptor pathways in which ZEB1, ZEB2, and SMAD are major players.

The protein–protein interaction networks for the *ZEB2* gene were obtained as shown in Figure 4. ZEB2 interacts with 24 other proteins and these interactions are predicted to impact gene expression and the binding of encoded proteins (see Figure 4). Seventeen of the twenty-four genes are predicted to show co-expression with ZEB2 protein binding, while ten genes are predicted to interact via protein binding. The two SMAD proteins, SMAD1 and SMAD3, which are shown to interact with ZEB2 via co-expression and binding, are main signal transducers for receptors of the transforming growth factor beta superfamily, critical for regulating cell development and growth. The encoded ZEB2 protein contains a SMD protein domain. Additionally, the ZEB2 protein is predicted to interact with four PAX proteins, two POU3F proteins, and two GATA proteins. These interactions have the potential to impact the ZEB2 protein’s functional pathways and interactions related to the TGF receptor for which ZEB1, ZEB2, and SMAD are major players.

### 2.5. ZEB2 Structural Protein Domains, Functions and Models

**Figure 5:**
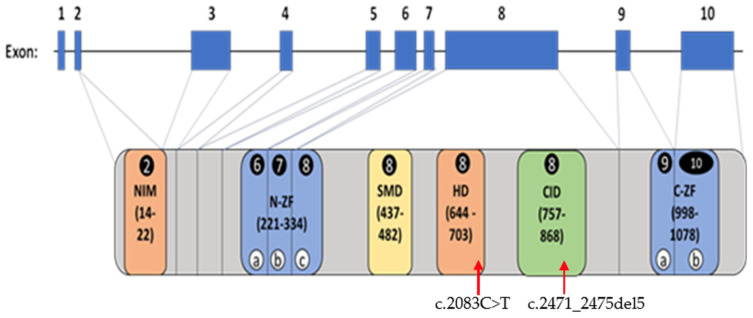
Schematic representation of *ZEB2* gene structure, exons, and codons listed and incorporated into each of the six protein domains. The protein domains and their corresponding exons are illustrated and modified from Zou et al. [13] such as NIM (nucleosome remodeling and deacetylase-interaction motif), N-ZF (N-terminal zinc finger cluster), SMD (SMAD-binding domain), HD (homeodomain), CID (CtBP-interacting domain), and C-ZF (C-terminal zinc finger cluster) modified from reference [13]. Exons coding the individual protein domains and domain regions are circled. The circled lowercase letters (a/b/c) found in both the N-ZF and C-ZF domains represent the regions coded by different exons (exon numbers circled). The most common gene variant (c.2083C>T) found in our study of 298 patients with MWS and the pathogenetic variant (c.2471_2475del5) found in our 5-year-old proband along with their locations in the HD and CID domains, respectively, are indicated by red arrows.

The ZEB2 protein domains and their corresponding exons are represented in Figure 5. As illustrated in this figure, the *ZEB2* gene consists of 10 exons, each of varying size, and encodes for six protein domains with specific functions and domain relationships described below.

**Figure 6:**
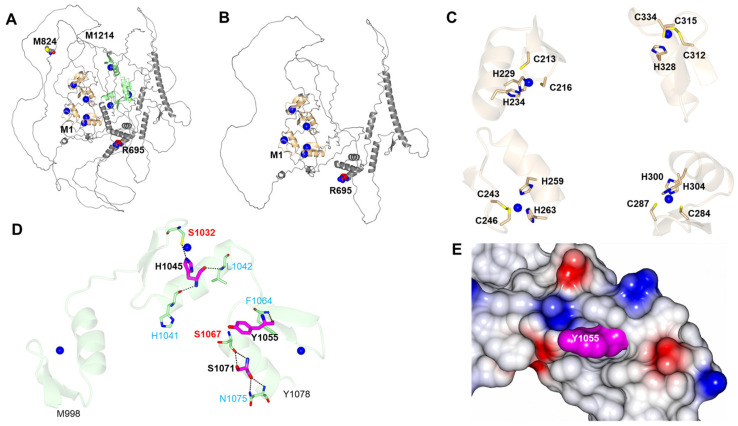
Predicted AlphaFold2 structure of ZEB2 (www.uniprot.org/uniprotkb/O60315/entry; accessed on 29 September 2023). (**A**) Full-length structure showing the N-terminal zinc finger domain (tan, L211-C334) and the C-terminal zinc finger domain (green, M998-Y1078) with codon positions at R695 and M824 with their predicted zinc ion binding sites drawn as blue spheres. M824 is a codon that is deleted in our case report of a 5-year-old female with a pathogenic variant c.2472_2475del5 in exon 8, while the R695 protein variant was the most common protein defect found in the 298 reported patients with MWS summarized in Table 1. (**B**) Same view as panel A but with residues after R695 omitted, resulting in a polypeptide lacking the C-terminal zinc finger domain. (**C**) N-terminal zinc finger domain shows the predicted zinc binding sites and coordination with ZEB2 residues. (**D**) C-terminal zinc finger domain shows predicted side chain hydrogen bond interactions (dashed lines) between H1045, Y1055, and S1071 missense ZEB2 changes reported by Ghoumid et al. [12] and other ZEB2 residues (red). Y1055 does not form interactions with the side chain -OH but does form a backbone hydrogen bond interaction with the backbone N-atom of F1064. Other backbone interactions with H1045 and Y1055 are indicated with blue text. (**E**) Electrostatic surface representation shows packing of Y1055 within a cleft of the ZEB2 C-terminal zinc finger domain.

A predicted model from a computer-based Alphafold2 program (Uniprot:O60315) was used to generate an altered ZEB2 protein structure from a truncated protein, while haploinsufficiency leads to a lower amount of protein resulting from non-sense mediated decay (NMD) of mRNA containing the abnormal gene defect. As shown below in Figure 6A, the ZEB2 protein is composed of many flexible/disordered regions that contain well-structured ZF domains. Residues after R695, the most common protein defect/site found in our studied MWS population, generated a stop codon in exon 8, which may result in a polypeptide lacking the C-terminal zinc finger (C-ZF) domain due to a truncated protein as shown in Figure 6B. Additionally, the N-ZF contains ZF domains predicted to coordinate zinc ions by the residues shown in Figure 6C.

The depicted H1045R, Y1055C, and S1071P variants in three patients with MWS were reported in 2013 by Ghoumid et al. [12] as missense mutations within the C-ZF protein domain. Interestingly, the H1045R and S1071P variants would likely result in the breakage of hydrogen bonds between the side chains and residues of S1032 and S1067 which could destabilize the ZF domains (Figure 6D). Although the side chain of Y1055 does not form hydrogen bond interactions with its side chain, this residue is tightly packed within a cleft and mutations would likely affect this interaction (Figure 6E).

The five base pair ZEB2 deletion in exon 8 at c.2471_2475del5 at the M824 residue in our proband would potentially impact codons of four of the six domains (about one-third of the N-ZF domain and all of the SMD, HD, and CID domains). Hence, this novel ZEB2 deletion variant could not be predicted or modeled due to a lack of computer protein access data information for modeling purposes with the involvement of such a large proportion of the ZEB2 protein. The Met824 residue is predicted to be absent leading to disruption of the Lys825 residue upstream of the C-ZF protein domain. This predicted mistranslated string of residues would result in the removal of the C-ZF domain. Therefore, the ZEB2 protein would be altered and/or presumably degraded in our proband, potentially impacting 24 other interacting genes found in gene–gene interaction and protein binding studies, as noted in Figure 4.

## 3. Discussion

### 3.1. Mowat–Wilson Syndrome (MWS) and Clinical Findings

Mowat–Wilson syndrome (MWS) is a rare autosomal dominant disorder caused by pathogenic variants in the *ZEB2* gene, located at chromosome 2q22.3. We identified about 300 known cases of MWS in the literature or unpublished databases. Most cases of MWS are sporadic heterozygous de novo occurrences with a low recurrence risk in siblings. We analyzed and summarized a wide range of ZEB2 defects including frameshift, nonsense, missense, and deletions or duplications. Those with the larger deletions tend to have more clinical severity.

Mowat–Wilson syndrome was first reported in 1998 [3,7] with multi-organ system involvement along with moderate-to-severe intellectual disability, speech delay, and microcephaly with brain anomalies. Seizures are often found with onset between several months to over 10 years of age. Specific facial dysmorphisms exist with a wide range of cardiac anomalies requiring intervention. Genitourinary and kidney anomalies are also present in about 50% of cases including undescended testicles, penile malformations, hydronephrosis, and kidney defects. Gastrointestinal problems include Hirschsprung disease in nearly one-half of reported patients accompanied by constipation. Other occasional abnormalities include long tapering toes and fingers, pes planus, nystagmus, cleft lip/palate, and pigmentary changes [3,7,8,9,10].

### 3.2. ZEB2 Gene Structure and Variants

The *ZEB2* gene consists of ten exons of different lengths and codes for six protein domains of variable sizes and functions. Exon 8 showed the largest number of variants and comprised 162 of the cases in our study population, followed by exon 6 with 27 variants. The large exon 8 accounts for about one-half of the *ZEB2* coding sequence and encodes the last zinc finger region of the N-ZF domain along with SMD, HD, and CID protein domains; hence, about 60% of the ZEB2 protein is coded by this exon, representing a similar proportion of the reported ZEB2 gene defects. Exon 8 is also involved in our 5-year-old proband with MWS.

Frameshift mutations or premature termination codons (PTCs) do lead to non-sense mediated decay (NMD), impacting protein production. NMD is a process that typically affects the mRNA of most genes with PTCs before the last exon, which leads to mRNA degradation with haploinsufficiency. It is likely that NMD does occur, affecting the amount of protein produced and causing the truncated protein to alter protein function in MWS as well as other genetic disorders. However, it is predicted that mutated transcripts of ZEB2 may not undergo non-sense mediated decay in MWS, but more research is needed [31].

We studied ZEB2 variant types and their locations within each exon. Nine of the ten exons, except exon 1, harbored damaging frameshift variants. Frameshift mutations occur when one or two bases are either inserted or deleted from a strand of DNA. This results in an aberrant protein impacting the protein function. This aligns with the fact that a frameshift defect was the most identified variant seen in our study, followed by nonsense mutations present in 6 of the 10 exons. A frameshift defect was seen in 134 of the 298 individuals [45%] reported with MWS. The second most frequent variant in our patient population was a nonsense defect seen in 112 patients [38%]. The third most frequent variant found was a large deletion in 21 individuals [7%]. These patients had a complete absence or deletion of the chromosome 2 region where the *ZEB2* gene is located. This was followed by missense variants in 11 individuals [4%]. Collectively, MWS is caused by haploinsufficiency of the autosomal dominant *ZEB2* gene function due to the heterozygous variants and copy number variations. Further studies to elucidate the precise molecular mechanisms for the pathogenicity of MWS are needed.

### 3.3. ZEB2 Gene Functions and Interactions

Recognized cellular components impacted by the *ZEB2* gene include the chromatin, cytosol, nucleolus, nucleus, and nucleoplasm, and impacted processes include the molecular function and disturbances of DNA-binding transcription factor activity via RNA polymerase and transcription repression, metal ion binding for zinc, and phosphatase regulatory activity. These derangements of the ZEB 2 protein may play a role in clinical outcomes, variability, and severity with early multi-organ development in MWS (www.uniprot.org/uniprotkb/O60315/entry, accessed on 1 November 2023). As described in our review, the *ZEB2* gene, when disturbed, could impact other interacting developmental genes by leading to a cascade of early perturbed biological processes and abnormal embryogenesis, requiring more research. The *ZEB2* gene functions as a transcriptional inhibitor by binding to specific DNA structure (5′-CACCT-3′) at gene promoters. It represses the transcription of cadherin 1 (*CDH1*; NM_004360.5) gene and cell adhesion required for the establishment and maintenance of epithelial cells during embryogenesis and adulthood which are important for brain and other organ development and function [32]. The mesenchyme homeobox 2 (*MEOX2*; NM_005924.5) gene [33] also interacts with ZEB2 as a homeobox gene involved in the regulation of anatomical development such as morphogenesis. Moreover, homeodomain proteins regulate gene expression and cell differentiation during early embryonic development and organogenesis including the heart, brain, and gut [34], which are involved in Mowat–Wilson syndrome. Gene functions and outcomes may also be altered by gene–gene or protein interactions or epistasis, whereby a single gene function may be impacted by interactions with other genes and disease processes [35].

### 3.4. Genes That Interact with ZEB2 and Potential Relationship with Mowat–Wilson Syndrome

The 24 genes recognized to interact with ZEB2 are shown in Figure 4. These gene–gene or protein interactions may play important roles in the derangement of multi-system embryogenesis and organogenesis. Among the interactive genes are five gene families (*SMAD*, *GATA*, *IRF*, *PAX*, and *POU3F*) represented with the *ZEB1* gene, presumably contributing to clinical findings seen in MWS. One of the interacting genes is a member of the *SMAD* gene family, *SMAD1* (*SMAD1*; NM_005900.3). The SMAD-related protein domain is one of the six domains found in the ZEB2 protein. The SMAD1 encoded protein is Involved in the downstream signaling pathway for bone morphogenic protein (BMP) subfamily members [36]. BMPs are known to play vital roles during the formation and maintenance of various organs disturbed in MWS, are members of TGF-β receptor signaling and are involved in skeletal dysplasia, DNA-binding transcription factor activity, and protein kinase binding. *SMAD3* (*SMAD*; NM_005902.4) targets genes involved in epithelial cells including cyclin-dependent kinase (CDK) inhibitors that generate a cytostatic response important for the regulation of muscle-specific genes [8]. The GATA-binding protein 1 (GATA1) interactive gene is X-linked and encodes the zinc finger DNA-binding transcription factor, which plays a critical role in the normal development of hematopoietic cells and is associated with thrombocytopenia, beta thalassemia, and dyserythropoietic anemia. The *GATA4* (*GATA4*; NM_001308093.3) interactive gene is associated with congenital heart disease, as seen in our 5-year-old female, and cardiac conduction, testicular development, and chromatin binding [37,38].

Other interactive genes include interferon regulatory factor 1 (*IRF1*: NM_002198.3) which is highly regulated in human vascular lesions and exhibits a growth inhibitory function in coronary artery smooth muscle, nitric oxide production, endothelial tissue, vascular intimal growth with healing, and pathophysiology of primary atherosclerosis [39]. The *IRF8* (*IRF8*; NM_002163.4) gene codes transcriptional agents of the interferon regulatory factor (IRF) family involved with conserved DNA-binding domains in the N-terminal regions and divergent C-terminal regions serving as regulatory sites, and in immunodeficiency [40].

The paired box 2 (*PAX2*; NM_000278.5) gene and other *PAX* family members are involved with mesenchyme to epithelium transition in renal development and related pathways for neural stem cells, lineage-specific markers, and Wnt/Hedgehog/Notch signaling [8]. For example, defects of the *PAX3* (*PAX3*: NM_181458.4) gene cause Waardenburg syndrome, involved with chromatin organization and binding, ectoderm differentiation, and craniofacial-spinal development with myogenesis, which are possibly important in MWS, as well [41]. The *PAX4* (*PAX4*; NM_001366110.1) gene encodes transcription factors that are essential for the formation of several tissues representing all germ layers and induced pluripotent stem cells with lineage-specific markers [42]. *PAX5* (*PAX5*; NM_016734.3) is a transcription factor gene essential for B-cell differentiation and other hematopoietic lineages and diseases [43]. The POU domain, class 3, transcription factor 1 (*POU3F1*; NM_002699.4) gene is associated with related pathways involved with nervous system development, mammalian neurogenesis, and myelination [8,44,45]; *POU3F2* (*POU3F2*; NM_005604.4) is involved with microphthalmia and melanoma, and *MECP2* (*MECP2*; NM_001110792.2) activity that is disturbed in Rett syndrome [8].

The STRING database (STRING.org) was used to study predicted protein–protein associations, networks, and functional enrichment analysis [46]. In this database, there are 10 significantly associated proteins with ZEB2 including CTBP1, CHURC1, ARHGAP31, TWIST1, TWIST2, SMAD3, CDH1, CDH2, HDAC1, and ZEB1. Only ZEB1 and SMAD3 are in common between this database and the Pathwayscommons.org database [32] depicted in Figure 4.

The C-terminal binding protein 1 (CTBP1) which interacts with the ZEB2 protein in the STRING database involves dehydrogenase activity and functions in brown adipose tissue differentiation [47]. The Churchill domain-containing 1 (CHURC1) protein is involved in the positive regulation of transcription with ubiquitous expression in multiple tissues [8]. The Rho GTPase-activating protein 31 (ARHGAP31) functions as a GTPase-activating protein (GAP) for RAC1 and CDC42 required for cell spreading, polarized lamellipodia formation, and cell migration. The Twist family transcription factor 1 or twist-related protein 1 (TWIST1) also acts as a transcriptional regulator, inhibits myogenesis, and represses the expression of pro-inflammatory cytokines such as TNFA and IL1B. It also regulates cranial suture patterning and fusion. The Twist-related protein 2 (TWIST2) binds to the E-box consensus sequence 5′-CANNTG-3′ and represses the expression of proinflammatory cytokines involved with glycogen storage and energy metabolism as well as inhibiting the premature differentiation of pre-osteoblasts during osteogenesis. The Mothers against decapentaplegic homolog 3, receptor-regulated (SMAD3) binds to the TRE element of the promotor of many genes regulated by TGF-β, working with the *SMAD4* (*SMAD4*: NM_005359.6) gene, playing a role in multiple organ development and wound healing. Both cadherin-1 (CDH1) and cadherin-2 (CDH2) are calcium-dependent cell adhesion proteins, specifically for neural stem cells, and mediate anchorage to ependymocytes during maturation. Histone deacetylase 1 (HDAC1) is responsible for the deacetylation of lysine residues on the N-terminal part of the core histones and is involved in epigenetic repression with an important role in transcriptional regulation, cell cycle progression, and developmental events. Lastly, the zinc finger E-box-binding homeobox 1 (*ZEB1*; NM_001174096.2) is directly related to the *ZEB2* gene causing Mowat–Wilson syndrome and acts as a transcriptional repressor by inhibiting interleukin-2 (*IL-2*; NM_000586.4) gene expression. It also represses the E-cadherin promoter and induces an epithelial mesenchymal transition that positively regulates neuronal differentiation and plays a role in neurogenesis, as similarly seen with ZEB2 [47,48].

### 3.5. ZEB2 Protein Domains and Functions

There are six protein domains within the *ZEB2* gene, and these negatively impact *ZEB2* function, if altered by gene variants. The NIM (nucleosome remodeling and deacetylase interaction motif) protein domain functions as one of the major chromatin remodeling complexes. The N-ZF (N-terminal zinc finger cluster) and C-ZF (C-terminal zinc finger cluster) are significant players in gene regulation and function [49]. Moreover, in Xenopus, the N-ZF domain was also found to have an important role in early stages of neural induction [50]. These two zinc finger clusters are responsible for functions such as ZEB2 binding to DNA. These DNA-binding proteins often work to pack and modify DNA or regulate gene expression and are therefore crucial for proper functioning for DNA binding in vitro [51]. The SMD (SMAD-binding domain) functions to mediate TGF-β signaling in metazoan embryo development and adult tissue regeneration and homeostasis [52], while HD (homeodomain) regulates the expression of other genes in development [53]. The CID (CtBP-interacting domain) functions to regulate transcription, predominantly as a corepressor in the nucleus [54] impeding transcription and translation. The CtBP-interacting domain (CID) is responsible for the direct interaction of ZEB2 with CtBPs found at repeated PLDLS-like motifs thought to make ZEB2 more efficient at transcriptional suppression. CtBPs on their own do not necessarily have the ability to bind DNA in a gene/promoter specific context but rely on the recruitment of DNA-binding transcription factors such as ZEB2 to function [48]. Additional research is required to further understand the role of these protein domains and their function in causing clinical variability among patients diagnosed with MWS. This research would impact diagnosis, clinical care, treatment, and surveillance as well as genetic counseling of first-degree family members.

## 4. Conclusions

In our study, we report on a 5-year-old female with features commonly seen in MWS and with a novel pathogenic heterozygous c.2471_2475del5 in exon 8 of the *ZEB2* gene. This frameshift defect presumably disrupts ZEB2 protein production, quantity, and quality, as the five base pair deletion in exon 8 would impact the coding of three protein domains (CID, HD, and SMD) and about one-third of the N-ZF domain. The novel defect directly affects the encoding of the CID domain. The CID domain plays a role as a transcriptional corepressor, the HD domain regulates the expression of developmental genes, the SMD domain regulates signaling protein and embryo development, while the N-ZF domain is involved in gene regulation. 

Computer literature and unreported databases were searched for keywords such as Mowat–Wilson, *ZEB2* gene and protein defects or variants, or clinical features and about 180 published reports were found including 298 patients. These sources were used to collect data regarding *ZEB2* gene variants, types, frequencies, and protein defects along with domain locations and functions. Frameshift variants followed by nonsense variants accounted for more than 90% of the *ZEB2* gene defects. We found that exon 8, as the largest exon, encodes at least three of the six protein domains of the *ZEB2* gene and accounts for 66% (198/298) of the variants identified. *ZEB2* gene-gene or protein interactions were studied, and 24 separate proteins were predicted to share molecular functions with protein binding effects on embryo development impacting craniofacial, spine, brain, cardiovascular, kidney and hematopoiesis. ZEB2 also plays a role in the conversion of neuroepithelial cells in early brain formation and as a mediator of trophoblast differentiation.

## Figures and Tables

**Table 1:** Review of Mowat–Wilson syndrome with *ZEB2* gene and protein variants.

*Our Study ID*	*Publication ID/* *(Patient Number)*	*ZEB2 Exon*	*ZEB2 Gene Variant or Defect*	*Protein Defect*	*Type of Genetic* *Defect*	ZEB2 *Protein Domain*
1	Mowat–Wilson Syndrome Foundation [MWSF]/(P1)	-	c.2180del	p.Leu727Tyrfs*7	Frameshift	-
2	MWSF/(P2)	8	c.2083C>T	p.Arg695Ter	Nonsense	HD
3	MWSF/(P3)	9	c.3002del	p.Cys1001LeufsX74	Frameshift	C-ZFa
4	MWSF/(P4)	8	c.2761C>T	p.Arg921*	Nonsense	-
5	MWSF/(P5)	9	c.3095G>A	p.Cys1032Tyr	Missense	C-ZFb
6	MWSF/(P6)	10	c.3213dup	p.Q1072AfsX52	Frameshift	C-ZFb
7	MWSF/(P7)	6	c.696C>G	p.Tyr232*	Nonsense	N-ZFa
8	MWSF/(P8)	6	c.805C>T	p.Q269X	Nonsense	N-ZFb
9	MWSF/(P9)	8	c.2061del	p.Phe687Leufs*2	Frameshift	HD
10	MWSF/(P10)	10	-	p.Tyr999*	Nonsense	C-ZFa
11	MWSF/(P11)	3	c.108del	p.E37fsX74	Frameshift	-
12	MWSF/(P12)	8	c.2721del	p.Thr908LeufsTer22	Frameshift	-
13	MWSF/(P13)	2	c.81_84dup	p.Asp29Leufs*2	Frameshift	-
14	MWSF/(P14)	8	c.2094C>A	p.Y698X	Nonsense	HD
15	MWSF/(P15)	6	c.763C>T	p.Q255X	Nonsense	N-ZFa
16	MWSF/(P16)	intron 3	c.331+1_331+2dup	-	Splicing	-
17	MWSF/(P17)	10	c.3533del	-	Deletion	-
18	MWSF/(P18)	10	c.3196C>T	p.His1066Tyr	Missense	C-ZFb
19	MWSF/(P19)	8	c.2083C>T	p.R695X	Nonsense	HD
20	MWSF/(P20)	8	c.2083C>T	p.R695X	Nonsense	HD
21	MWSF/(P21)	8	c.2367del	-	Frameshift	CID
22	MWSF/(P22)	6	c.674C>A	p.S225X	Nonsense	N-ZFa
23	MWSF/(P23)	8	c.909_910ins	p.H304Ffs*5	Frameshift	N-ZFc
24	MWSF/(P24)	8	c.1795del	p.His599MetfsX8	Frameshift	-
25	MWSF/(P25)	2	c.31del	p.Arg11Glyfs*16	Frameshift	-
26	MWSF/(P26)	8	c.1571_1572ins	p.Ser524Argfs*4	Frameshift	-
27	MWSF/(P27)	1–10	6.2 Mb deletion Ch2q22.1-q22.3	-	Chromosome deletion	-
28	MWSF/(P28)	8	c.1956C>A	p.Y652X	Nonsense	HD
29	MWSF/(P29)	1–10	6.9 Mb deletion Ch2q22.1-q22.3	-	Chromosome deletion	-
30	MWSF/(P30)	4	c.357_358del	p.Met120GlyfsX11	Frameshift	-
31	MWSF/(P31)	-	144 kb deletion Ch2q22.3	-	Chromosome deletion	-
32	MWSF/(P32)	10	c.3212_3215dup	p.Gln1072HisfsTer53	Frameshift	C-ZFb
33	[13] Zou et al., 2020/(P1)	3	c.290G>A	p.Trp97X	Nonsense	-
34	Zou et al., 2020/(P2)	8	c.1067_1068ins	p.Val357Aspfs*15	Frameshift	-
35	Zou et al., 2020/(P3)	8	c.2761C>T	p.Arg921X	Nonsense	-
36	Zou et al., 2020/(P4)	8	c.3214C>T	p.Gln1072X	Nonsense	C-ZFb
37	[14] Hu et al., 2020/(P1)	3	c.250G>T	p.E84*	Nonsense	-
38	[15] Ho et al., 2020/(P1)	8&9	*ZEB2* gene Exons 8 and 9 deletion	-	Deletion	-
39	Ho et al., 2020/(P2)	8	c.1472_c.1473ins	p.Met491llefs*9	Small insertion, Frameshift	-
40	Ho et al., 2020/(P3)	8	c.2083C>T	p.Arg695*	Nonsense	HD
41	Ho et al., 2020/(P4)	8	c.1387del	p.Val463Phefs*24	Frameshift	SMD
42	Ho et al., 2020/(P5)	8	c.2646del	p.Val883Cysfs*4	Small deletion, Frameshift	-
43	Ho et al., 2020/(P6)	3	c.189del	p.Ser64Valfs*11	Small deletion, Frameshift	-
44	Ho et al., 2020/(P7)	3	c.189del	p.Ser64Valfs*11	Small deletion, Frameshift	-
45	Ho et al., 2020/(P8)	1–10	*ZEB2* gene Exons 1-10 deletion	-	Deletion	-
46	Ho et al., 2020/(P9)	9	c1297C>T	p.Gln433*	Nonsense	-
47	Ho et al., 2020/(P10)	10	c.3335del	p.Tyr1112Cysfs*128	Small deletion, Frameshift	-
48	Ho et al., 2020/(P11)	1–10	*ZEB2* gene Exons 1-10 deletion	-	Deletion	-
49	Ho et al., 2020/(P12)	7	c.857_858del	p.Glu286Valfs*8	Small deletion, Frameshift	N-ZFb
50	Ho et al., 2020/(P13)	3	c.291G>A	p.Trp97*	Nonsense	-
51	Ho et al., 2020/(P14)	8	c.2865C>A	p.Tyr955*	Nonsense	-
52	Ho et al., 2020/(P15)	9	c.169delins	p.Leu565*	Small indel, Frameshift	-
53	[16] Wenger et al., 2014/(P1)	8	-	p.R695X	Nonsense	HD
54	Wenger et al., 2014/(P2)	8	-	p.Q384X	Nonsense	-
55	Wenger et al., 2014/(P3)	8	-	p.G1182KfsX59	Frameshift	-
56	Wenger et al., 2014/(P4)	5	-	p.E181Rfs211X	Frameshift	-
57	Wenger et al., 2014/(P5)	9	-	p.F1008C	Missense	C-ZFa
58	Wenger et al., 2014/(P6)	7	c.808-1G>T	-	Splicing	N-ZF
59	Wenger et al., 2014/(P7)	10	-	p.Ser1071Pro	Missense	C-ZFb
60	Wenger et al., 2014/(P8)	8	-	p.L894FfsX53	Frameshift	-
61	Wenger et al., 2014/(P9)	8	-	p.S434Vfs7X	Frameshift	-
62	Wenger et al., 2014/(P10)	8	-	p.R695X	Nonsense	HD
63	Wenger et al., 2014/(P11)	8	-	p.R695X	Nonsense	HD
64	Wenger et al., 2014/(P12)	8	-	p.R695X	Nonsense	HD
65	Wenger et al., 2014/(P13)	8	-	p.M476WfsX11	Frameshift	-
66	Wenger et al., 2014/(P14)	8	-	p.P906LfsX24	Frameshift	-
67	Wenger et al., 2014/(P15)	6	-	p.Q209X	Nonsense	-
68	Wenger et al., 2014/(P16)	8	-	p.V627SfsX4	Frameshift	-
69	Wenger et al., 2014/(P17)	9	-	p.S1011AfsX53	Frameshift	C-ZFa
70	Wenger et al., 2014/(P18)	6	-	p.R218RfsX21	Frameshift	-
71	Wenger et al., 2014/(P19)	8	-	p.V621AfsX25	Frameshift	-
72	Wenger et al., 2014/(P20)	1–10	-	-	Whole gene deletion	-
73	Wenger et al., 2014/(P21)	8	-	p.S359TfsX3	Frameshift	-
74	Wenger et al., 2014/(P22)	8	-	p.R695X	Nonsense	HD
75	Wenger et al., 2014/(P23)	8	-	p.Q962X	Nonsense	-
76	Wenger et al., 2014/(P24)	8	-	p.A907LfsX23	Frameshift	-
77	Wenger et al., 2014/(P25)	8	-	p.G351VfsX19	Frameshift	-
78	Wenger et al., 2014/(P26)	5	-	p.E181RfsX211	Frameshift	-
79	Wenger et al., 2014/(P27)	8	c.1224del	-	Deletion	-
80	Wenger et al., 2014/(P28)	8	-	p.I390LfsX6 & p.L388F	Frameshift	-
81	[6] Baxter et al., 2017/(P1)	1&2	-	-	Partial duplication	-
82	[17] Mundhofir et al., 2012/(P1)	8	c.1965C>G	p.Tyr652∗	Nonsense	HD
83	[18] Murray et al., 2015/(P1)	8	c.2083C>T	p.R695X	Nonsense	HD
84	[19] Wang et al., 2019/(P1)	7	c.904C>T	p.R302X	Nonsense	N-ZFc
85	Wang et al., 2019/(P2)	6	c.756C>A	p.Y252X	Nonsense	N-ZFa
86	Wang et al., 2019/(P3)	8	c.2761C>T	p.R921X	Nonsense	-
87	[20] Yamada et al., 2014/(P1)	3	c.259G>T	p.E87X	Nonsense	-
88	Yamada et al., 2014/(P2)	3	c.259G>T	p.E87X	Nonsense	-
89	Yamada et al., 2014/(P3)	3	c.259G>T	p.E87X	Nonsense	-
90	Yamada et al., 2014 (P4)	7	c.811C>T	p.Q271X	Nonsense	N-ZFb
91	Yamada et al., 2014/(P5)	7	c.904C>T	p.R302X	Nonsense	N-ZFc
92	Yamada et al., 2014/(P6)	8	c.936C>A	p.C312X	Nonsense	N-ZFc
93	Yamada et al., 2014/(P7)	8	c.1027C>T	p.R343X	Nonsense	-
94	Yamada et al., 2014/(P8)	8	c.1027C>T	p.R343X	Nonsense	-
95	Yamada et al., 2014/(P9)	8	c.1027C>T	p.R343X	Nonsense	-
96	Yamada et al., 2014/(P10)	8	c.1298C>T	p.Q433X	Nonsense	-
97	Yamada et al., 2014/(P11)	8	c.1489C>T	p.Q497X	Nonsense	-
98	Yamada et al., 2014/(P12)	8	c.1645A>T	p.R549X	Nonsense	-
99	Yamada et al.,2014/(P13)	8	c.1825G>T	p.E609X	Nonsense	-
100	Yamada et al., 2014/(P14)	8	c.2083C>T	p.R695X	Nonsense	HD
101	Yamada et al., 2014/(P15)	8	c.2083C>T	p.R695X	Nonsense	HD
102	Yamada et al., 2014/(P16)	8	c.2083C>T	p.R695X	Nonsense	HD
103	Yamada et al., 2014/(P17)	8	c.2083C>T	p.R695X	Nonsense	HD
104	Yamada et al., 2014/(P18)	8	c.2083C>T	p.R695X	Nonsense	HD
105	Yamada et al., 2014/(P19)	8	c.2083C>T	p.R695X	Nonsense	HD
106	Yamada et al., 2014/(P20)	8	c.2083C>T	p.R695X	Nonsense	HD
107	Yamada et al., 2014/(P21)	8	c.2083C>T	p.R695X	Nonsense	HD
108	Yamada et al., 2014/(P22)	8	c.2083C>T	p.R695X	Nonsense	HD
109	Yamada et al., 2014/(P23)	8	c.2083C>T	p.R695X	Nonsense	HD
110	Yamada et al., 2014/(P24)	8	c.2083C>T	p.R695X	Nonsense	HD
111	Yamada et al., 2014/(P25)	8	c.2083C>T	p.R695X	Nonsense	HD
112	Yamada et al., 2014/(P26)	8	c.2399C>G	p.S800X	Nonsense	CID
113	Yamada et al., 2014/(P27)	8	c.2615C>G	p.S872X	Nonsense	-
114	Yamada et al., 2014/(P28)	8	c.2761C>T	p.R921X	Nonsense	-
115	Yamada et al., 2014/(P29)	8	c.2761C>T	p.R921X	Nonsense	-
116	Yamada et al., 2014/(P1)	3	c.162_164del	p.P55Lfs*20	Frameshift	-
117	Yamada et al., 2014/(P2)	3	c.175_182del	p.T60Sfs*3	Frameshift	-
118	Yamada et al., 2014/(P3)	3	c270_272del	p.G91Vfs*17	Frameshift	-
119	Yamada et al., 2014/(P4)	3	c.311_312dup	p.A105Sfs*16	Frameshift	-
120	Yamada et al., 2014/(P5)	5	c.459_460del	p.E154Rfs*58	Frameshift	-
121	Yamada et al., 2014/(P6)	6	c.635_638dup	p.P214Lfs*26	Frameshift	-
122	Yamada et al., 2014/(P7)	6	c.647del	p.C216Sfs*8	Frameshift	-
123	Yamada et al., 2014/(P8)	6	c.759_760dup	p.Q255Pfs*8	Frameshift	N-ZFa
124	Yamada et al., 2014/(P9)	7	c.852_855del	p.T285Rfs*9	Frameshift	N-ZFb
125	Yamada et al., 2014/(P10)	7	c.855_858del	p.E286Vfs*8	Frameshift	N-ZFb
126	Yamada et al., 2014/(P11)	7	c.862_863del	p.G288Afs*10	Frameshift	N-ZFb
127	Yamada et al., 2014/(P12)	8	c.1169ins	p.I390Tfs*41	Frameshift	-
128	Yamada et al., 2014/(P13)	8	c.1169_1170del	p.T392Qfs*4	Frameshift	-
129	Yamada et al., 2014/(P14)	8	c.1174_1178del	p.T392Nfs*3	Frameshift	-
130	Yamada et al., 2014/(P15)	8	c.1176del	p.E393Nfs*3	Frameshift	-
131	Yamada et al., 2014/(P16)	8	c.1212_1213del	p.A405Lfs*12	Frameshift	-
132	Yamada et al., 2014/(P17)	8	c.1268_1273del	p.S424Lfs*2	Frameshift	-
133	Yamada et al., 2014/(P18)	8	c.1280_1286del7ins	p.G427Dfs*2	Frameshift	-
134	Yamada et al., 2014/(P19)	8	c.1334_1337dup	p.L447Ffs*9	Frameshift	SMD
135	Yamada et al., 2014/(P20)	8	c.1395_1408del14ins	p.Q465Hfs*9	Frameshift	SMD
136	Yamada et al., 2014/(P21)	8	c.1417del	p.R473Gfs*14	Frameshift	SMD
137	Yamada et al., 2014/(P22)	8	c.1417del	p.R473Gfs*14	Frameshift	SMD
138	Yamada et al., 2014/(P23)	8	c.1421_1426dup	p.M476Nfs*6	Frameshift	SMD
139	Yamada et al., 2014/(P24)	8	c.1492_1493del	p.P498Lfs*18	Frameshift	-
140	Yamada et al., 2014/(P25)	8	c.1534_1535del	p.G512Vfs*4	Frameshift	-
141	Yamada et al., 2014/(P26)	8	c.1822del	p.E608Kfs*13	Frameshift	-
142	Yamada et al., 2014/(P27)	8	c.1966_1967del	p.M656Vfs*17	Frameshift	HD
143	Yamada et al., 2014/(P28)	8	c.2178_2180del	p.L727Ifs*28	Frameshift	-
144	Yamada et al., 2014/(P29)	8	c.2254dup	p.T752Nfs*4	Frameshift	-
145	Yamada et al., 2014/(P30)	8	c. 2282del	p.T761Kfs*26	Frameshift	CID
146	Yamada et al., 2014/(P31)	8	c.2349_2351dup	p.S784Ffs*11	Frameshift	CID
147	Yamada et al., 2014/(P32)	8	c.2579del	p.L860Rfs*3	Frameshift	CID
148	Yamada et al., 2014/(P33)	8	c.2740_2743dup	p.S916Dfs*34	Frameshift	-
149	Yamada et al., 2014/(P34)	10	c.3608_3614del	p.D1204Rfs*29	Frameshift	-
150	[21] Tronina et al., 2023/(P1)	6	-	p.Gln694Ter	Missense	HD
151	[22] Jakubiak et al., 2021/(P1)	8	c.1027C > T	p.R343*	Nonsense	-
152	Jakubiak et al., 2021/(P2)	1–10	-	-	Deletion	-
153	Jakubiak et al., 2021/(P3)	6	c.648C > A	p.C216*	Nonsense	-
154	Jakubiak et al., 2021/(P4)	10	*ZEB2* gene Exon 10 deletion	-	Deletion	-
155	Jakubiak et al., 2021/(P5)	8	c.1946del	p.I649Tfs*17	Frameshift	HD
156	Jakubiak et al., 2021/(P6)	6	c.607ins	p.Thr203IlefsTer37	Frameshift	-
157	Jakubiak et al., 2021/(P7)	4	c.399_400dup	p.Thr134IlefsTer3	Frameshift	-
158	Jakubiak et al., 2021/(P8)	8	c.1276T > A	p.Leu426Ile	Missense	-
159	Jakubiak et al., 2021/(P9)	6	c.696C > G	p.Y232*	Nonsense	N-ZFa
160	Jakubiak et al., 2021/(P10)	-	8 Mb deletion 2q22.3q23.3	-	Chromosome deletion	-
161	Jakubiak et al., 2021/(P11)	1–10	-	-	Deletion	-
162	Jakubiak et al., 2021/(P12)	3–10	-	-	Deletion	-
163	Jakubiak et al., 2021/(P13)	7	c.857-858del	p.Glu286ValfsTer8	Frameshift	N-ZFb
164	Jakubiak et al., 2021/(P14)	6	c.607ins	p.Thr203IlefsTer37	Frameshift	-
165	Jakubiak et al., 2021/(P15)	8	c.1445T > G	p.Leu482*	Nonsense	SMD
166	Jakubiak et al., 2021/(P16)	3	c.84T > G	p.Tyr28*	Nonsense	-
167	Jakubiak et al., 2021/(P17)	8	c.1421-1426del	p.Gln474_Met476delins	Insertion, deletion	SMD
168	Jakubiak et al., 2021/(P18)	8	c.2230A > G	p.Ile744Val	Missense	-
169	Jakubiak et al., 2021/(P19)	10	c.3202G > T	p.Gly1068Cys	Missense	C-ZFb
170	Jakubiak et al., 2021/(P20)	8	c.2087_2088del	Lys696Serfs*24	Frameshift	HD
171	Jakubiak et al., 2021/(P21)	8	c.2073G > A	p.Trp691Ter	Nonsense	HD
172	Jakubiak et al.,2021/(P22)	-	-	-	Deletion	-
173	Jakubiak et al., 2021/(P23)	8	c.2562_2564del	p.N855Lfs*3	Frameshift	CID
174	Jakubiak et al., 2021/(P24)	8	c.1177dup	p.E393Gfs*7	Frameshift	-
175	Jakubiak et al., 2021/(P25)	8	c.1437_1440del	p.H304Qfs*3	Frameshift	N-ZFc
176	Jakubiak et al., 2021/(P26)	8	c.2083C > T	p.Arg695Ter	Nonsense	HD
177	Jakubiak et al., 2021/(P27)	8	c.2083C > T	p.Arg695Ter	Nonsense	HD
178	Jakubiak et al., 2021/(P28)	3–10	258.2 kb deletion	-	Chromosome deletion	-
179	[23] Refaat et al., 2021/(P1)	-	2.27 Mb deletion	-	Chromosome deletion	-
180	[24] Musaad et al., 2022/(P1)	-	Chr2:145161574del	-	Frameshift	-
181	[25] Pachajoa et al., 2022/(P1)	8	c.2761C>T	p.Arg921Ter	Nonsense	-
182	[26] Wu et al., 2022/(P1)	8	c.2417del	p.Phe807Serfs*11	Frameshift	CID
183	Wu et al., 2022/(P2)	8	c.1200T>A	p.Tyr400X	Nonsense	-
184	Wu et al., 2022/(P3)	8	c.1027C>T	p.Arg343X	Nonsense	-
185	Wu et al., 2022/(P4)	8	c.2621del	p.Asn874Ilefs*12	Frameshift	-
186	Wu et al., 2022/(P5)	8	c.2456C>G	p.Ser819X	Nonsense	CID
187	Wu et al., 2022/(P6)	8	c.2002del	p.Glu668Serfs*8	Frameshift	HD
188	Wu et al., 2022/(P7)	2–10	Chr2:145147017-145274917del	-	Large deletion	-
189	Wu et al., 2022/(P8)	5	c.492_517del	p.Glu164Aspfs*9	Frameshift	-
190	Wu et al., 2022/(P9)	6	c.779dup	p.Met260Ilefs*19	Frameshift	N-ZFa
191	Wu et al., 2022/(P10)	1–10	chr2:141213978-148010654del	-	Large deletion	-
192	Wu et al., 2022/(P11)	8	c.2083C>T	p.Arg695X	Nonsense	HD
193	Wu et al., 2022/(P12)	1–10	chr2:145000000-145351228 del	-	Large deletion	-
194	Wu et al., 2022/(P13)	7	c.904C>T	p.Arg302X	Nonsense	N-ZFc
195	Wu et al., 2022/(P14)	8	c.2712del	p.Pro906Leufs*24	Frameshift	-
196	Wu et al., 2022/(P15)	8	c.2670_2677del	p.Ala891Phefs*55	Frameshift	-
197	Wu et al., 2022/(P16)	8	c.2177_2180del	p.Ser726Tyrfs*7	Frameshift	-
198	Wu et al., 2022/(P17)	1–10	chr2:138434153-145285163 del	-	Large Deletion	-
199	Wu et al., 2022/(P18)	8	c.2851C>T	p.Gln951X	Nonsense	-
200	Wu et al., 2022/(P19)	8	c.1426dup	p.Met476Asnfs*5	Frameshift	SMD
201	Wu et al., 2022/(P20)	8	c.2761C>T	p.Arg921X	Nonsense	-
202	Wu et al., 2022/(P21)	8	c.1027C>T	p.Arg343X	Nonsense	-
203	Wu et al., 2022/(P22)	8	c.1106_1115delins	p.Leu369X	Nonsense	-
204	[27] Fu et al., 2022/(P1)	8	c.2136del	p.Lys713Serfs*3	Frameshift	-
205	Fu et al., 2022/(P2)	8	c.2740del	p. Gln914Argfs*16	Frameshift	-
206	Fu et al., 2022/(P3)	7	c.808-2del	-	Splicing	N-ZFb
207	[28] Wei et al., 2021/(P1)	8	c.1137_1146del	p.S380Nfs*13	Deletion	-
208	[29] Şenbil et al., 2021/(P1)	6	c.646dup	p.Cys216LeufsTer23	Frameshift	-
209	[30] Ivanoski et al., 2018/(P1)	6	c.805C>T	p.Q269*	Nonsense	N-ZFb
210	Ivanoski et al., 2018/(P2)	1–10	*ZEB2* gene deletion	-	Large deletion	-
211	Ivanoski et al., 2018/(P3)	1–10	7.3 Mb deletion Ch2q22.1q22.3	-	Large deletion	-
212	Ivanoski et al., 2018/(P4)	8	c.1381C>T	p.Q461*	Nonsense	SMD
213	Ivanoski et al., 2018/(P5)	8	c.1052_1057delins	p.G351Vfs*19	Small deletion, Frameshift	-
214	Ivanoski et al., 2018/(P6)	6	c.696C>G	p.Y232*	Nonsense	N-ZFa
215	Ivanoski et al., 2018/(P7)	8	c.1073_1122delins	p.S359Tfs*3	Small indel, Frameshift	-
216	Ivanoski et al., 2018/(P8)	3	c.310C>T	p.Q104*	Nonsense	-
217	Ivanoski et al., 2018/(P9)	1–10	2.6 Mb deletion Ch2q22.2q22.3	-	Large deletion	-
218	Ivanoski et al., 2018/(P10)	8	c.2718del	p.A907Lfs*23	Small deletion, Frameshift	-
219	Ivanoski et al., 2018/(P11)	8	c.2180T>A	p.L727*	Nonsense	-
220	Ivanoski et al., 2018/(P12)	1–10	*ZEB2* gene deletion	-	Large deletion	-
221	Ivanoski et al., 2018/(P13)	8	c.1381C>T	p.Q461*	Nonsense	SMD
222	Ivanoski et al., 2018/(P14)	8	c.2083C>T	p.R695*	Nonsense	HD
223	Ivanoski et al., 2018/(P15)	5	c.460G>T	p.E154*	Nonsense	-
224	Ivanoski et al., 2018/(P16)	8	c.2083C>T	p.R695*	Nonsense	HD
225	Ivanoski et al., 2018/(P17)	8	c.1426dup	p.M476Nfs*6	Small insertion, Frameshift	SMD
226	Ivanoski et al., 2018/(P18)	1–10	4.6 Mb deletion Ch2q22q22.3	-	Large deletion	-
227	Ivanoski et al., 2018/(P19)	8	c.2682_2687delins	p.L894Ffs*36	Small indel, Frameshift	-
228	Ivanoski et al., 2018/(P20)	3	c.274G>T	p.G92*	Nonsense	-
229	Ivanoski et al., 2018/(P21)	8	c.2053C>T	p.Q685*	Nonsense	HD
230	Ivanoski et al., 2018/(P23)	9	c.3031del	p.S1011Afs*64	Small deletion, Frameshift	C-ZFa
231	Ivanoski et al., 2018/(P24)	8	c.2227del	p.S743Lfs*2	Small deletion, Frameshift	-
232	Ivanoski et al., 2018/(P25)	5	c.460del	p.E154Rfs*58	Small deletion, Frameshift	-
233	Ivanoski et al., 2018/(P26)	6	c.625C>T	p.Q209*	Nonsense	-
234	Ivanoski et al., 2018/(P27)	7	c.817del	p.L273*	Nonsense	N-ZFb
235	Ivanoski et al., 2018/(P28)	8	c.1635_1636ins	p.D546Lfs*11	Small insertion, Frameshift	-
236	Ivanoski et al., 2018/(P29)	3	c.310C>T	p.Q104*	Nonsense	-
237	Ivanoski et al., 2018/(P30)	3	c.310C>T	p.Q104*	Nonsense	-
238	Ivanoski et al., 2018/(P31)	8	c.2701C>T	p.Q901*	Nonsense	-
239	Ivanoski et al., 2018/(P32)	8	c.2083C>T	p.R695*	Nonsense	HD
240	Ivanoski et al., 2018/(P34)	8	c.2718del	p.A907Lfs*23	Small deletion, Frameshift	-
241	Ivanoski et al., 2018/(P35)	8	c.2317_2318del	p.E773Kfs*8	Small deletion, Frameshift	CID
242	Ivanoski et al., 2018/(P36)	1–10	0.6 Mb deletion Ch2q22.2	-	Large deletion	-
243	Ivanoski et al., 2018/(P37)	3	c.264_267del	p.I88Mfs*19	Small deletion, Frameshift	-
244	Ivanoski et al., 2018/(P38)	8	c.1541del	p.P414Rfs*2	Small deletion, Frameshift	-
245	Ivanoski et al., 2018/(P39)	8	c.2856del	p.R953Efs*24	Small deletion, Frameshift	-
246	Ivanoski et al., 2018/(P40)	8	c.2254dup	p.Y752Nfs*4	Small insertion, Frameshift	-
247	Ivanoski et al., 2018/(P41)	8	c.930C>A	p.Y310*	Nonsense	N-ZFc
248	Ivanoski et al., 2018/(P42)	8&9	c.917_3067del	p.E307_G1023del	Deletion, in-frame	N-ZFc C-ZFa
249	Ivanoski et al., 2018/(P43)	8	c.975C>A	p.Y325*	Nonsense	N-ZFc
250	Ivanoski et al., 2018/(P44)	8	c.2083C>T	p.R695*	Nonsense	HD
251	Ivanoski et al., 2018/(P45)	6	c.691dup	p.Y232Vfs*7	Frameshift	N-ZFa
252	Ivanoski et al., 2018/(P46)	8	c.2083C>T	p.R695*	Nonsense	HD
253	Ivanoski et al., 2018/(P47)	7	c.901del	p.L301Cfs*37	Small deletion, Frameshift	N-ZFc
254	Ivanoski et al., 2018/(P48)	1–10	*ZEB2* gene deletion	-	Large deletion	-
255	Ivanoski et al., 2018/(P49)	3	c.81_84dup	p.D29Lfs*2	Small insertion, Frameshift	-
256	Ivanoski et al., 2018/(P50)	8	c.1202dup	p.K401Ifs*17	Small insertion, Frameshift	-
257	Ivanoski et al., 2018/(P51)	6	c.648C>A	p.C216*	Nonsense	-
258	Ivanoski et al., 2018/(P52)	8	c.1910C>G	p.S637*	Nonsense	-
259	Ivanoski et al., 2018/(P53)	8	c.2713del	p.P906Lfs*24	Small deletion, Frameshift	-
260	Ivanoski et al., 2018/(P54)	8	c.2083C>T	p.R695*	Nonsense	HD
261	Ivanoski et al., 2018/(P55)	5	c.540del	p.E181Rfs*31	Small deletion, Frameshift	-
262	Ivanoski et al., 2018/(P56)	6	c.609del	p.P204Qfs*9	Small deletion, Frameshift	-
263	Ivanoski et al., 2018/(P57)	5	c.477_484del	p.H159Qfs*10	Small deletion, Frameshift	-
264	Ivanoski et al., 2018/(P58)	8	c.2083C>T	p.R695*	Nonsense	HD
265	Ivanoski et al., 2018/(P59)	5–8	c.403_2887del	p.V135Gfs*5	Frameshift	-
266	Ivanoski et al., 2018/(P60)	8	c.2083C>T	p.R695*	Nonsense	HD
267	Ivanoski et al., 2018/(P61)	1–10	16.7 Mb deletion Ch2q21.1q22.3	-	Large deletion	-
268	Ivanoski et al., 2018/(P62)	6	c.653_654ins	p.G219Pfs*21	Small insertion, Frameshift	-
269	Ivanoski et al., 2018/(P63)	8	c.1851del	p.H617Qfs*4	Small deletion, Frameshift	-
270	Ivanoski et al., 2018/(P64)	4	c.389_390dup	p.T134Lfs*3	Small insertion, Frameshift	-
271	Ivanoski et al., 2018/(P65)	8	c.2076del	p.F692Lfs*24	Small deletion, Frameshift	HD
272	Ivanoski et al., 2018/(P66)	8	c.2083C>T	p.R695*	Nonsense	HD
273	Ivanoski et al., 2018/(P67)	7	c.823C>T	p.Q275*	Nonsense	N-ZFb
274	Ivanoski et al., 2018/(P68)	8	c.1313del	p.H438Pfs*2	Small deletion, Frameshift	SMD
275	Ivanoski et al., 2018/(P69)	1&2	*ZEB2* gene Exons 1 and 2 deletion	-	Large deletion	-
276	Ivanoski et al., 2018/(P70)	8	c.1027C>T	p.R343*	Nonsense	-
277	Ivanoski et al., 2018/(P71)	6	c.648C>A	p.C216*	Nonsense	-
278	Ivanoski et al., 2018/(P72)	1–10	*ZEB2* gene deletion	-	Large deletion	-
279	Ivanoski et al., 2018/(P73)	8	c.1381C>T	p.Q461*	Nonsense	SMD
280	Ivanoski et al., 2018/(P74)	10	*ZEB2* gene Exon 10 deletion (3′UTR c.3642+750)	-	Large deletion	-
281	Ivanoski et al., 2018/(P75)	8	c.1946del	p.I649Tfs*17	Small deletion, Frameshift	HD
282	Ivanoski et al., 2018/(P76)	2	c.32dup	p.R11Pfs*9	Small insertion, Frameshift	-
283	Ivanoski et al., 2018/(P77)	6	c.761del	p.Q255Sfs*7	Small deletion, Frameshift	N-ZFa
284	Ivanoski et al., 2018/(P78)	8	c.1553del	p.H518Lfs*26	Small deletion, Frameshift	-
285	Ivanoski et al., 2018/(P79)	8	c.936C>A	p.C312*	Nonsense	N-ZFc
286	Ivanoski et al., 2018/(P80)	3	c.187_228dup	p.A77Lfs*13	Small insertion, Frameshift	-
287	Ivanoski et al., 2018/(P81)	8	c.1150C>T	p.Q384*	Nonsense	-
288	Ivanoski et al., 2018/(P82)	5	c.553_554ins	p.R185Lfs*28	Small insertion, Frameshift	-
289	Ivanoski et al., 2018/(P83)	7	c.857_858del	p.E286Vfs*8	Small deletion, Frameshift	N-ZFb
290	Ivanoski et al., 2018/(P84)	10	c.3567_3568ins	p.M1190Pfs*52	Small insertion, Frameshift	-
291	Ivanoski et al., 2018/(P85)	8	c.2677del	p.P893Lfs*37	Small deletion, Frameshift	-
292	Ivanoski et al., 2018/(P86)	8	c.2372del	p.T791Nfs*26	Small deletion, Frameshift	CID
293	Ivanoski et al., 2018/(P87)	6	c.715del	p.E239Rfs*23	Small deletion, Frameshift	N-ZFa
294	Ivanoski et al., 2018/(P88)	IVS1	c.-69-2A>C	p.M1_N24delins	Splicing	-
295	Ivanoski et al., 2018/(P89)	8	c.1578_1579delins	p.D527Tfs*17	Small indel, Frameshift	-
296	[12] Ghoumid et al., 2013/(P1)	10	c.3134A>G	p.His1045Arg	Missense	C-ZFb
297	Ghoumid et al., 2013/(P2)	10	c.3164A>G	p.Tyr1055Cys	Missense	C-ZFb
298	Ghoumid et al., 2013/(P3)	10	c.3211T>C	p.Ser1071Pro	Missense	C-ZFb

Ter, X or * represent stop codons at the time of publication; N-ZF = N-terminal zinc finger clusters domain (221–334); SMD = SMAD-binding domain (437–482); IVS1 = intervening sequence; HD = homeodomain-like domain (644–703); CID = CtBP-interacting domain (757–868); C-ZF = C-terminal zinc finger clusters domain (998–1078). Protein domain regions coded by exons are represented based on codon location within the domains (e.g., exon 9 codes for C-ZFa and exon 10 codes for C-ZFb). References are listed in brackets [6,12,13,14,15,16,17,18,19,20,21,22,23,24,25,26,27,28,29,30].

## Data Availability

Data are contained within the article.
